# Objectively measured active transportation to school and other destinations among 10–13 year olds

**DOI:** 10.1186/s12966-017-0634-4

**Published:** 2018-01-19

**Authors:** Gillian C. Williams, Michael M. Borghese, Ian Janssen

**Affiliations:** 1 Department of Public Health Sciences, Queen’s University, Kingston, ON K7L 3N6 Canada; 2 School of Kinesiology and Health Studies, Queen’s University, Kingston, ON K7L 3N6 Canada

**Keywords:** Child, Physical activity, Transportation, Walking, Bicycling

## Abstract

**Background:**

Descriptive data on active transportation in children focuses on the trip to school and has relied on subjective reports. The purpose of this study was to use objective measures to describe total active transportation and active transportation to common destinations within children.

**Methods:**

This was a descriptive study of 388 children aged 10–13 years from Kingston, Ontario, Canada. Participants wore a Garmin GPS watch during waking hours for seven days. Personal Activity Measurement Location System software used the GPS data to identify trips, time spent in each trip and the trip modality (walking, bicycle or vehicle). Google Maps software was used to identify trip destinations.

**Results:**

A total of 8875 trips were identified. Most (69%) trips were made by vehicle; 25% were made by walking and 6% by bicycle. Mean time spent in active transportation was 10.3 min/day (95% CI: 7.4, 14.5). Time spent in active transportation was higher for boys (12.1 min/day [95% CI: 8.8, 17.0) than for girls (8.5 min/day [95% CI: 6.1, 12.0]) and increased from 7.7 min/day (95% CI: 5.5, 11.1) at age 10 to 14.3 min/day (95% CI: 10.3, 19.9) at age 13. Time spent in active transportation was lower in the winter by comparison to the other seasons. The four most common active transportation destinations were the participant’s home, school, other people’s homes, and parks or greenspace with 69%, 39%, 37% and 32% of participants walking or bicycling to these destinations at least once over the 7-day measurement period.

**Conclusion:**

Over 65% of trips made and time spent travelling occurred in a vehicle. When active transportation was used, the most common destinations were home, school, other people’s homes, and parks.

## Background

Physical activity benefits a child’s physical, mental, and social health [1, 2]. Active transportation, such as walking or cycling, can be an important source of physical activity [3–5]. Active transportation research among young people has focused on the trip to school. This research indicates that there has been a global decline in active transportation to school in recent decades [6], and that the proportion of students who actively commute to school varies greatly across countries (e.g., 80% in Zimbabwe to 13% in the United States) [7, 8].

Because previous research has focused on travel to school, childrens’ active transportation habits to other destinations are largely unknown [9]. Furthermore, existing research has largely relied upon self- or parent-reported active transportation data [10]. Objectively measured data, research on total active transportation, and research on travel destinations other than school is needed. The recent availability of wearable Global Position System (GPS) loggers and Geographic Information System (GIS) software for the measurement of active transportation provides an opportunity to address these research gaps and limitations. Indeed, active transporation studies have starting using these objective measures [5, 11–15].

The purpose of this study was to use objective measures to describe total active transportation and active transportation to common travel destinations among a sample of 10–13 year olds from Kingston, Ontario, Canada. Consideration was given to the age and sex of the participants, the season in which active transportation was measured, and the travel mode (i.e., walking, bicycle, or vehicle).

## Methods

### Participants

This was a descriptive study of 10–13 years olds from Kingston, Ontario, Canada. Kingston is a mid-sized city (population 123,798) with a modest population density (273 people per km^2^) [16]. Approximately 15% of the population live in the urban core, 63% live in the suburbs, and the remaining 22% live in exurban and rural areas [17]. There is a large season variation in temperature in Kingston, with daily average temperatures ranging from −7.0 °C in January to 21.5 °C in July [18].

A sample of 458 children were recruited from the approximately 5000 eligible children [16]. Eligibility criteria were being aged 10–13 years, living and attending school in the city of Kingston, and ability to communicate in either of Canada’s official languages (English or French). Data were collected between January 2015 and December 2016 and were balanced across the four seasons, age, sex, and proportional of the population of 10–13 year olds residing in each of the city’s 12 electoral districts [16]. Children were compensated $40 for completing the study. Participants and a parent/guardian provided consent. The study was approved by the General Research Ethics Board at Queen’s University.

### Active transportation data collection and cleaning

Participants were provided with verbal and written instructions on how to wear a Garmin Forerunner 220 GPS watch (Garmin Ltd., Schaffhausen, Switzerland). Participants wore the watch for 7 consecutive days. They were instructed to put on the watch shortly after waking, to turn on the GPS logger function, and to continue wearing the watch until bedtime, at which time they removed the watched so that its battery could be charged overnight. During waking hours the watch recorded their longitude and latitude coordinates approximately every 2 s to 2 min depending on satellite signal availability. The GPS data were downloaded from the watch using Garmin Connect software (Garmin Ltd., Schaffhausen, Switzerland) and then exported to Personal Activity and Location Measurement System (PALMS) software. The software filtered extreme observations and interpolating invalid GPS points caused by brief signal loss. PALMS provides a validated method for processing GPS data to objectively measure time spent in different transportation modes [19]. PALMS identified trips based on sequential GPS points spanning ≥100 m with a speed ≥1 km/h over ≥3 min in duration. The software allowed for pauses in travel of up to 3 min to account for traffic lights and other brief stops. PALMS classified the modality of each trip as vehicle, bicycle, or walking based on travel speed. Trips with a 90th percentile of speed ≥25 km/h were classified as vehicle trips, trips with a 90th percentile of speed ≥10 km/h and <25 km/h were classified as bicycle trips, and trips with a 90th percentile of speed ≥1 km/h and <10 km/h were classified as walking trips.

After PALMS software identified the trips and the modality of each trip, we used Google Fusion Tables (Google, Mountain View, California, USA) to visually inspect the GPS points of each trip which allowed us to remove false positive trips and identify the destination of each trip. False positives trips refer to trips identified by PALMS that reflect other movements such as outdoor play in the school yard. The 6595 trips that were identified by PALMS that occurred solely within a specific location (e.g., the school yard at recess) were classified as false positive trips and were deleted during the cleaning process.

During the cleaning process we identified the destination of each trip by examining its endpoint using satellite and street view images in Google Maps (Google, Mountain View, California, USA). Destinations were categorized based on the descriptions described in Table 1. Note that trips to school were categorized based on the date/time of travel and whether or not it was the participant’s school. Trips to school in the morning on school days were categorized in the “school” category. Trips leaving school at the end of the school day were captured in the “home” destination or wherever the participant travelled after school. Trips to school on non-school days, during the middle of the school day (e.g., field trips, leaving school property at lunch), and after school hours (e.g., returning to school for an event) were categorized as “school day trips and trips to school for non-curriculum purposes.”

**Table 1:** Description of trip destinations

Destination	Description
Home	The participant’s primary or secondary residence.
Other homes	Homes other than the primary or secondary residence.
School bus stop	Locations where participants travelled to so that they could be picked up by a school bus.
School	Trips to the participant’s school immediately prior to the start of the school day.
School day trips and trips to school for non-curriculum purposes	Trips to school during the school day (e.g., returning to school from a field trip or medical appointment), or returning to school in the evenings or on non-school days (e.g., for an after-school event, to play on school grounds on the weekend).
Parks or greenspace	City parks, undeveloped greenspace, or conservation areas.
Recreation facilities	Government and commercial recreation and sport facilities (e.g., arenas, sports fields, gymnastics club).
Retail locations	Supermarkets, grocery stores, convenience stores, gas stations, big box stores, strip malls, stand-alone stores, downtown stores, and shopping malls.
Food service locations	Fast food restaurants, full service restaurants, coffee shops, and dessert and ice cream shops.
Community locations	Entertainment or cultural facilities, churches or other places of worship, community centres, libraries.
Other	Health care facilities, other schools (not the school where the participant was enrolled), transit stations, police station etc.

Note that active transportation trips with the same starting and end destination, such as hikes or walking around the neighbourhood, were also removed during the cleaning process as they were considered walking or bicycling for leisure rather than active transportation to a specific destination.

Next, the data were imported into SAS 9.4 statistical software (SAS Institute, Cary NC) for further processing and to calculate the number of minutes of active transportation. During this process we deleted data from days with <10 h of GPS data (i.e., invalid days) and participants (*n* = 70) with <4 valid days. This step is consistent with data processing done with accelerometer measures of physical activity [20, 21]. The SAS program then determined the total number of trips, the total duration of each trip (based on the times that the GPS points of the trip start and end points were recorded), and total daily time spent in trips separately by trip modality and destination.

The reliability of the protocol used to clean the GPS data and identify trip destinations was determined by repeating all data cleaning steps for 50 participants in triplicate. One observer cleaned the data twice and a second observer cleaned the data a third time. Mean minutes/day in active transportation were consistent across all three cleaning scenarios: 14.8 (95% CI: 12.3, 17.3) for the initial clean by observer 1, 15.9 (95% CI: 13.5, 18.3) for the second cleaning by observer 1, and 14.7 (95% CI: 12.7, 16.8) for the clean by the second observer. Intra-rater percent agreement for destinations was 90% and inter-rater agreement was 88%.

### Sociodemographic and covariate data

Sociodemographic characteristics including age, sex, and race (white, other) of the participant, family structure (number of parents and siblings in the household), family income, and parental education were obtained from a parent survey. Season of participation was categorized based on equinox and solstice dates. The body mass index was calculated based on heights and weights measured using a stadiometer and digital scale, respectively. The World Health Organization references were used to categorize participants as having a non-overweight (thin + normal), overweight, or obese body mass index [22].

### Statistical analysis

Data were analyzed using SAS 9.4. Transportation behaviours were presented in several ways: the number of trips made, the proportion of participants who travelled to different destinations over the 7 day measurement period, the proportion of trips that were made by walking or bicycling, and average daily time spent traveling using different travel modes. An initial examination of the data revealed that 10% of participants did not have any active transportation trips and that the active transportation data could not be transformed to follow a normal distribution. Therefore, a two-part modelling strategy was used to estimate average min/day of active transportation. This is a strategy commonly used for health-care cost data which is similarly distributed [23, 24]. In the first part of the two-part model, logistic regression was used to model the probability of the presence of any active transportation minutes. In the second part of the two-part model, a generalized linear model was used to model active transportation minutes in participants who engaged in any active transportation. To fit this model the proc genmod procedure was used with a logarithmic link function and gamma distribution. All models were adjusted for age, sex and season. Other covariates (race, number of parents in household, number of siblings in household, family income, and parental education) were entered in the model using backwards selection methods and were retained based on a significance level of *p* < 0.1 in either the logistic regression or general linear model. The mean daily time spent engaged in  active transportation was calculated by multiplying the estimates from the two models together. ANOVA and Bonferroni post hoc comparison tests were used to compare active transportation by age, sex, and season for destinations with a daily average > 1 min.

## Results

### Descriptive characteristics

Seventy participants were removed during data processing due to insufficient GPS data. This left a final sample of 388. Among these 388 participants, 5% had 4 valid days of GPS data, 14% had 5 valid days, 31% had 6 valid days, and 49% had 7 valid days. On valid days there was an average of 13.7 h (95% CI: 13.6, 13.8) of GPS data. Descriptive characteristics of the participants are in Table 2. Approximately half were boys (51%) and participants were evenly distributed across ages and seasons. The majority were white (87%) and lived in a dual parent household (86%).

**Table 2:** Participant characteristics

Characteristic	Number	Percent
Sex		
Boy	197	50.8
Girl	191	49.2
Age (years)		
10	94	24.2
11	97	25.0
12	101	26.0
13	96	24.7
BMI		
Not overweight	287	74.0
Overweight	62	16.0
Obese	39	10.1
Race		
White	336	86.6
Other	52	13.4
Season of Participation		
Winter	101	26.0
Spring	99	25.5
Fall	90	23.2
Summer	98	25.3
Number of Parents in Household		
Dual Parent	335	86.3
Single Parent	50	12.9
No response	3	0.8
Number of Siblings in Household		
0	47	12.1
1	196	50.5
2	101	26.0
3+	44	11.4
Family Income ($ CDN per year)		
50,000	57	14.7
50,001–100,000	112	28.9
> 100,000	176	45.4
No response	43	11.1
Parental Education		
High school or less	32	8.3
2-year college	115	29.6
4-year college/university	241	62.1

### Proportion of trips made using active transportation

After removal of 6595 false positive trips during data cleaning, a total of 8875 trips remained. Most (69%) of these trips were made by vehicle; 25% were made by walking and 6% by bicycle. Thirty-eight (10%) participants did not have any active trips and 249 (64%) did not have any bicycle trips.

Table 3 provides information on trips to different destinations. In the total sample there were a total of 366 trips made for each 100 observation days. The most common travel destinations were the participant’s own home (119 trips per 100 days), trips to school to start the school day (54 trips per 100 days), retail locations (45 trips per 100 days), and other people’s homes (32 trips per 100 days). The most common active transportation destinations were the participant’s own home, their own school, other people’s homes, and parks or greenspace with 69%, 39%, 37% and 32% of participants walking or bicycling to these destinations at least once over the 7-day measurement period. Approximately 65% of all active transportation trips were made to one of these four destinations. Fewer than 25% of participants made an active transportation trip to a retail location, restaurant, recreation facility, community centre, or other community locations (e.g., place of worship, arts or entertainment venue) and <25% of trips to these locations were made using active transportation.

**Table 3:** Descriptive information on trips and trips to different destinations within the entire sample (*n* = 388)

Destination	% (95% CI) who travelled to destination using any mode	% (95% CI) who travelled to destination using active travel	# of trips to destination (per 100 person days)	% of trips to destination made by walking	% of trips to destination made by bicycle	Mean (95% CI) minutes/day of active travel to destination
All destinations	100	90.0 (87.2, 93.2)	366	31.1	6.3	10.3 (7.4, 14.5)^a,b,c^
Home	100	69.3 (64.7, 73.9)	119	25.2	6.6	3.1 (2.1, 4.5)^a,b,c^
Other people’s homes	71.9 (67.4, 76.4)	36.6 (31.8, 41.4)	32	24.3	11.0	1.0 (0.7, 1.5)^a^
Bus stop	20.6 (16.6, 24.7)	14.2 (10.7, 17.7)	16	42.8	0.5	0.2 (0.1, 0.3)^a^
School to start school day	82.5 (78.7, 86.3)	38.7 (33.8, 43.5)	54	29.2	7.1	2.1 (1.5, 3.0)^a,b^
Other trips to school	33.0 (28.3, 37.7)	23.5 (19.2, 27.7)	10	57.2	13.5	0.7 (0.5, 1.0)^a^
Parks or greenspace	46.4 (41.4, 51.4)	32.2 (27.5, 36.9)	16	11.7	14.8	1.0 (0.6, 1.5)^a,b^
Recreation facilities	68.3 (63.6, 72.9)	17.0 (13.3, 20.8)	30	13.1	3.4	0.5 (0.3, 0.8)^a^
Retail locations	84.3 (80.6, 87.9)	24.7 (20.4, 29.1)	45	12.7	3.0	0.8 (0.5, 1.1)^a^
Food service locations	53.1 (48.1, 58.1)	16.0 (12.3, 19.6)	15	22.4	2.2	0.5 (0.3, 0.8)^a^
Community locations	47.4 (42.4, 52.4)	11.3 (8.2, 14.5)	13	13.8	3.8	0.4 (0.2, 0.6)^a^
Other	46.6 (41.7, 51.6)	15.2 (11.6, 18.8)	17	19.8	5.4	0.3 (0.2, 0.5)^a^

^a^Adjusted for sex, age, season

^b^Adjusted for number of siblings in household

^c^Adjusted for single or dual parent household

### Time spent in active transportation

On average, participants spent 10.3 min/day (95% CI: 7.4, 14.5) participating in active transportation. The average daily time spent walking and bicycling for travel were 9.4 min/day (95% CI: 7.3, 12.2) and 1.8 min/day (95% CI: 1.2, 2.8), respectively. This is in contrast with a mean of 57.4 min/day (95% CI: 47.6, 69.2) of vehicle travel. The participant’s own home was the destination with the highest minutes of active transportation with a daily average of 3.1 (95% CI: 2.1, 4.5) minutes, followed by school to start the school day (2.1 min [95% CI: 1.5, 3.0]) and other people’s homes (1.0 min [95% CI: 0.7, 1.5]). Mean min/day of active transportation was <1 min for all other destinations.

Table 4 shows the average daily minutes of active transportation to destinations with a mean > 1 min/day according to age, sex and season. Mean daily minutes of active transportation for boys (12.1 min/day [95% CI: 8.8, 17.0]) was significantly higher than for girls (8.5 min/day [95% CI: 6.1, 12.0]). Time spent in active transportation increased from 7.7 min/day (95% CI: 5.5, 11.1) at age 10 to 14.3 min/day (95% CI: 10.3, 19.9) at age 13. Time spent in active transportation was statistically lower in the winter by comparison to the other 3 seasons. Similar patterns to those described for total active transportation were observed for active transportation to the most common active transportation destinations (Table 3).

**Table 4:** Sex, age, and season difference in minutes/day spent in active travel by travel destination for those destinations where the mean active travel time was >1 min/day

Characteristic	Travel destination			
	All destinations^h,i,j^	Participant’s own home^h,i,j^	Other people’s homes^h^	School to start school day^h,i^
Total (*n* = 388)	10.3 (7.4, 14.5)	3.1 (2.1, 4.5)	1.0 (0.7, 1.5)	2.1 (1.5, 3.0)
Sex				
Boys (*n* = 197)	12.1 (8.8, 17.0)	3.7 (2.6, 5.4)	1.2 (0.8, 1.7)	2.2 (1.6, 3.1)
Girls (*n* = 191)	8.5 (6.1, 12.0)^a^	2.4 (1.6, 3.5)^a^	0.8 (0.6, 1.3)	1.9 (1.3, 2.7)
Age				
10 (*n* = 94)	7.7 (5.5, 11.1)	2.2 (1.5, 3.4)	0.7 (0.5, 1.1)	2.0 (1.4, 2.7)
11 (*n* = 97)	9.7 (6.9, 13.7)	2.8 (2.0, 4.1)	1.1 (0.7, 1.5)	1.7 (1.2, 2.4)
12 (*n* = 101)	9.6 (7.0, 13.4)	3.1 (2.2, 4.5)	1.0 (0.7, 1.6)	2.0 (1.4, 2.9)
13 (*n* = 96)	14.3 (10.3, 19.9)^b,c,d^	4.0 (2.8, 5.8)^b,c^	1.2 (0.8, 1.7)	2.5 (1.8, 3.6)
Season				
Winter (*n* = 101)	6.4 (4.7, 8.9)	2.1 (1.4, 3.1)	0.4 (0.3, 0.6)	1.6 (1.1, 2.2)
Spring (*n* = 99)	12.6 (9.2, 17.3)^e^	3.6 (2.6, 5.2)^e^	1.2 (0.8, 1.7)^e^	2.3 (1.6, 3.4)
Summer (*n* = 90)	12.2 (8.7, 17.2)^e^	3.0 (2.1, 4.4)^e^	1.5 (1.0, 2.1)^e^	1.5 (1.0, 2.1)
Fall (*n* = 98)	10.4 (7.3, 15.1)^e^	3.5 (2.4, 5.1)^e^	1.0 (0.7, 1.4)	2.7 (2.0, 3.9)^g^

^a^Significantly different from boys (*p* < 0.05)

^b^Significantly different from 10 year olds (*p* < 0.05)

^c^Significantly different from 11 year olds (*p* < 0.05)

^d^Significantly different from 12 year olds (*p* < 0.05

^e^Significantly different from winter (*p* < 0.05)

^f^Significantly different from spring (*p* < 0.05)

^g^Significantly different from summer (*p* < 0.05)

^h^Adjusted for sex, age, season

^i^Adjusted for number of siblings

^j^Adjusted for single or dual parent household

## Discussion

This study objectively measured active transportation levels and destinations among a diverse sample of 10–13 year olds from Kingston, Ontario, Canada. Overall, 10% of participants did not have any active transportation trips over the course of the week and only 37% of trips were made using active transportation. Participants spent 10 min/day engaged in active transportation. Home, school and other people’s homes were the most common active travel destinations. Significant differences in active transportation were found by age, sex and season.

Only a few prior studies have described objectively measured active transportation data among children and youth [5, 11–15]. In our study, 90% of participants engaged in active transportation at least once over the course of the week, which is comparable to a study of 12–16 year old Americans where 77% engaged in active transportation over a week [11]. In that American study 43% of the trips were made using active transportation, which is similar to the value of 37% found in our study, but much lower than the value of 74% observed among 14–18 year olds in Portugal [5]. The average time spent in active transportation is also higher in children from other European countries than in our Canadian sample. Specifically, the average daily time spent in active transportation was 22.5 min/day in a sample of 10–13 year old Belgian children [15] and 19.0 min/day in a sample of 11–16 year old Danish youth [14]. This is consistent with self-reported active transportation data which consistently show that by comparison to North America, a greater proportion of children from European countries walk or bicycle to school [10, 25, 26]. The differences in active travel between North America and Europe reflect policies on pedestrian safety, cultural norms and the compact nature of European cities that allow for short trips.

In terms of travel destinations, parental reported data on a sample of 7–12 year olds from Canada and the United States suggested that 28% walked or bicycled to someone else’s home at least three times per week and that 21% walked or bicycled to a park at least 3 times per week [27]. In our study of 10–13 year olds, 37% and 32% walked or bicycled to these destinations at least once in the week-long data collection period. We also found that 39% of participants had at least one active trip to their school to start the school day. This is in line with self- or parental-reported estimates in 5- to 17-year-olds in Canada: 42% use exclusively active transportation or a combination of active and passive transportation to travel to and from their school [9]. It is important to note that trips home from school were not categorized as school trips in our study as the categorization was based on the destination and not starting point of the trip. Therefore, we likely did not capture all students who use active transportation from school because the proportion of children who walk or bicycle home from school in the afternoon is about 10% higher than the proportion who walk or bicycle to school in the morning [28].

Our findings that active transportation was higher in boys than girls and increased from age 10 to 13 are consistent with what has been found in the literature based on questionnaire measures. Boys have consistently been found to engage in more active transportation than girls [29, 30]. Active transportation has been found to increase with age, peak during adolescence then decline [30–32]. In contrast with our results, where there was clear seasonal variation in active transportation, previous studies conducted in Ontario, Canada have not found an effect of the seasons on active transportation to school [30, 33]. However, these studies only considered data collected over the course of a school year or less.

The findings of our study have implications for public health practice. Specifically, our findings suggest that interventions should consider active transportation to destinations other than school, the focal point of almost all active travel interventions in children [34], and be cognisant of sex disparities and the influence of season on active transportation. The average of 11 min/day of active transportation in this study represents only 18% of the target needed to meet the public health recommendation of 60 min/day of moderate-to-vigorous physical activity [35]. Other data from the study sample that was not presented in this paper indicates that they accumulated an average of 54 min/day of moderate-to-vigorous activity, which is within a few minutes of the moderate-to-vigorous activity levels observed in a nationally representative of Canadian children [36]. Collectively, these observations suggest that even modest increases in active transportation would result in a substantial increase in the proportion of children meeting public health recommendations for physical activity.

Our findings also have implications for the measurement of active transportation in research settings. Approximately 40% of trips originally identified by the PALMS software were false positive trips. Play in the school yard during recess or leisure trips (e.g., walking around the neighbourhood with the same starting point and destination) are examples of physical activities that were commonly identified as trips by PALMS software and which we needed to reclassify after the automated PALMS processing steps were completed. Future research is needed to determine the most accurate and most feasible ways to process the GPS data post-PALMS.

There are some limitations of this study. First, 15% of participants did not have adequate GPS data to be included in the analysis. Although there were no significant differences in the descriptive characteristics of those included and those excluded from the analyses, it is possible that their active transportation differed. Second, trips <100 m long and lasting <3 min were not captured by PALMS software. Therefore, short trips, such as to a nearby house or bus stop, were missed. Third, based on existing precedence in the physical activity field [21], participants could have as few as 4 days of valid data (i.e., ≥10 h of GPS wear time) to be included in the analyses. That would have led to misclassification had these 4–7 days did not reflect their habitual active transportation. Finally, these results are limited to 10–13 year olds from Kingston, Ontario, Canada.

## Conclusion

The use of objective measures to capture active transportation to a variety of destinations among children fills major calls for research in the active transportation field [9]. In this study of 10–13 year olds, approximately 1 in every 3 trips was made using active transportation. Children spent an average of 11 min/day walking or bicycling vs. 57 min/day travelling in a motorized vehicle. Home, school and other people’s homes were the destinations with the most active transportation minutes. Future research should continue to use objective measures but consider a greater age range as well as greater geographic diversity.

## Abbreviations

GIS: Geographic information systems
GPS: Global position system
